# Dr. Lowry, on Gout

**Published:** 1805-04-01

**Authors:** Stephen Lowry

**Affiliations:** Falmouth


### *To the Editors of the Medical and Physical Journal.*

Gentlemen,

The latter part of the eighteenth century will be ever recorded as the æra, when medicine had arrived to such a perfection as entitles it to the highest rank among the other branches of philosophy, and reflects a lustre on British practitioners not to be equalled in all Europe. Within these few years our attention has been called to some of the greatest discoveries that ever adorned the healing art.

The

The prospect of the annihilation of the small-pox: The wonderful effects that have been produced by the digitalis, not only in phthisis pulmonalis and confirmed dropsy, but in pneumonia and other diseases: The astonishing success that has attended the use of cold affusion in typhus, in tetanus, and other spasmodic diseases: The effects of the muriate of lime and barytes in that emphatically styled opprobrium medicorum, scrofula; all proclaim this.

But, perhaps, there is no circumstance in the Annals of Medicine that has excited more admiration in the profession, and caused more joy to individuals, than the cure of Gout. This Herculean disease has hitherto baffled all the attempts of physicians, not only to cure it, but even to alleviate its symptoms or protract its return; this was reserved for the present day, to join in the train of the other once opprobria medicorum, to crown the whole, and to give the name, "The Æra of Medicine."

But while we so justly admire the attempts that have lately been made by some of our own country, to throw new light upon the subject; amongst these, Mr. Mantal's paper in your extensively useful Journal for December last, deserves a high tribute of praise. None amongst your numerous Correspondents have ever taken up the subject, or discussed the merits of that excellent Treatise on Gout, and the use of the bark in that disease, written originally in Latin by Prof. Tavares, and which Dr. Adams has been so kind as to give us in English, through the medium of your Journal. The only reason I conclude of this neglect is, that we are too apt to overlook foreign publications, even of more enlightened countries than Portugal, seeing our own island stands so high in the medical world. And even here also I confess we have a proof of this; for although I can never sufficiently thank the Archiatros of Portugal and commend his treatise, yet I believe we shall find that one of our own countrymen, Dr. Kentish, in a treatise written about fourteen years since, will have the honour of priority in respect to his views of the nature of the disease, and of the use of the bark in the cure of it; but as it has been so amply confirmed by the Professor, both in his own case and in the cases of others, we are highly indebted to him; and if you will give me leave, I will add my testimony to the truth of it in my own case also.

As well as I can remember, the first attack I had of gout was in the instep, in the autumn of 1782, before I had reached my twenty-fifth year; there was no external inflammation, inflammation, and very little pain except on moving, and in a few days it entirely left me. I felt nothing more of it till the month of July in the next year, when I was seized with a severe pain in the great toe of my right foot. The paroxysm, though severe, was very short; four or five days I believe put an end to it. Although I have little doubt that the disease is hereditary in me, yet I believe that at this time it was brought into action by certain causes, which would have produced gout in subjects where it was not so. Early disappointments, severe losses, with their attendants, anxiety, vexation and care, joined with the fatigue, watching and cold, to which medical men are subject, and producing a train of dyspeptic symptoms, are sufficient causes, without the aid of dissipation, to produce gout; but though I had from other motives than that of prevention, quitted my former mode of living very soon after this last paroxysm, yet, in the latter end of 1784, it returned, and continued as before. Thus it did four other years following, without affecting any other part than the feet, and without any material derangement of the system. In October, 1789, notwithstanding my regular mode of living, which I have continued ever since, it attacked the knee and right hand; and in January, 1791, after suffering much from the duties of my profession, I was seized with a most severe paroxysm, which affected almost every joint; this lasted a fortnight, and just as it was over, before I was able to quit my room, I was under the necessity of visiting a patient in a critical situation, which immediately brought on a relapse that confined me a fortnight longer. From this time, despairing of a remedy, I became dispirited, and was obliged to relinquish a considerable part of my practice. Till this time it was only once in the year that I should have an attack; but after this it would be more frequent, and almost every joint in rotation affected: and for these last ten years I know not how many paroxysms I have had in each year; but generally three more distinct than the others; one, as the rain sets in, in the autumn; another every year but the present since 1797, about the beginning of January; and the other in one of the rainy months in the spring. To such a sufferer, how welcome was the account which Prof. Tavares gave of the use of the bark, and which happily came to my hand before the autumnal paroxysm came on. This happened on Wednesday the 17th of October last, about ten days after the rain sat in, when, after several days indisposition, I was seized with a severe attack in the right shoulder; the pain increased towards night with with great violence, and gave me every reason to expect a general attack. I passed a very painful night, and by the morning the pain extended to the elbow; it was now I determined to try the bark. From something I had eaten the day before I had two lax motions early in the morning, so that I judged any previous purgative unnecessary. My stomach was quite relieved, and I was well prepared for it. I began at ten o'clock on Thursday morning, and took two scruples only every two hours, or as often as my stomach would bear it, mixed with a few grains of pulv. arom. and two tea spoons full of brandy. I passed the night comfortably, and almost free from pain. The support and comfort I felt from the medicine rendered the use of wine and other stimuli, which I had been accustomed to take, almost unnecessary. I was so well on Friday as to conclude the whole was over; but in the afternoon the bark affected my bowels, and produced two or three evacuations; I was immediately seized in the hand, and it became severe. This I considered simply to arise from the bark losing its influence on the system by this unwished for effect. I then took about ten drops of laudanum with the next dose, and fifteen at night without the powder; this produced the desired effect. I suffered from my hand three hours pain only, and this much more tolerable than usual; and on Sunday a perfect crisis took place. I could not sufficiently express my feeling on the occasion, frequently saying with Gotfs. Held, "Cortex peruv. in podagra remedium est divinum;" and I thought

"The joy half lost, because not sooner found."

I have reason to believe the attack would have been general, because for these last ten years I have scarcely ever been seized in the hand without suffering in every part, and because I felt, as it were, a faint impression of the pain in all those joints that were usually affected in the progress of the disease. In a few days I was able to quit my room; and on the 29th I was so well recovered as to visit a patient without suffering a relapse, as was generally the case; but this happened about a month after, and gave me a further and clearer proof of the efficacy of this remedy. The shoulder not having quite recovered its strength, and using it inadvertently, by a much greater exertion than usual I brought on the pain; and being obliged to go out the next day, (Wednesday, November 28) and suffering also from a blow on my knee, I was threatened with another severe paroxysm. On Saturday the the pain left the shoulder, and threatened to attack the knee and feet. I instantly took a sufficient quantity of elect. sennæ, to produce two or three evacuations. On Sunday the pain seized the knee that had been bruised, and was rather severe; I then began the bark; and after taking only half an ounce, the pain and all the other symptoms left me entirely, and more completely than at first, which, I suppose, agreeably to Prof. Tavares, was owing to the previous laxative. From this time to the present day I have enjoyed better health than I have at this season for several years; and notwithstanding the winter has been, unusually severe, and every arthritic in the neighbourhood confined by the disease, I have been able to go out at all times, being more bold than formerly, knowing the remedy is at hand. From this I think I might say, with a little variation, with Virgil,

O fortunati . . . . . . bona si sua norint
Arthritici.

The remarks I have made are as follow:

The fit has always been made up of distinct paroxysms and remissions, and it has generally been ten days before any thing like a crisis has taken place. The usual paroxysms are about ten o'clock A. M. and seven P. M. The rigor, succeeding heat, and perspiration resemble more a fit of ague than any other disease. The attack has almost always been in the rain months. I consider it to be the offspring of debility, as abstinence and fatigue will invariably produce it in me; and the evening paroxysm is always lessened in proportion as I have been able to take some solid food at dinner. I found this so essential to me, that I have endeavoured to get an appetite by taking some wine, or spirits and water, about an hour before; and from not accustoming myself much to stimuli in health, I find the greatest benefit arise from their use in this particular during the whole gouty paroxysm. This being the case, I was more easily prevailed on to try the bark, especially as it has been given in some cases as food. I found it to be so in reality; and I am persuaded it may be taken in much larger doses if there be occasion. The cautions I observed were to guard against symptoms of indigestion on the one hand, and against its purgative effects on the other. Omitting it soon after meals and the last thing at night, and taking some aromatic tincture with it, prevented the one, and a few drops tinct. opii invariably prevented the other. I consider that regular good living, avoiding every thing that will occasion indirect debility, and as much exercise on horseback as can be taken without fatigue, avoiding suppers, and keeping early hours, the best methods of preventing a return of the paroxysm. I have observed, that after every fit, for these last ten years, I have been subject to a relapse twice or three times, or till the disease had worn itself out, or, more properly, till I had gained strength enough to resist it; how much this resembles ague! But this has not been the case now, as I was not so much weakened by its long continuance. Yet still there are not wanting those who oppose even this method of curing the disease. Apoplexy and hæmoptysis are held up as the dire effects. Since my remembrance, the cure of ague also was to be purchased at the great expence of liver complaints, dropsies, and obstructions of various kinds; but Prof. Tavares has been eight years a living proof to the contrary; and I look forward to the time when this prejudice will be so far removed as it is now in regard to ague.

From the attack invariably beginning with rigor and other symptoms of fever, and from the effects of the bark, I have considered the inflammation on the extremities as the effect of fever; in the same way as in the small-pox, measles, and other exanthemata, the eruption is generally in proportion to the fever, and our aim being principally directed to the cure of it by cold; might not a general exposure to cold air, on the first approach of the paroxysm, be of service in the gout also? Every one knows that very frequently a paroxysm of gout is preceded by symptoms resembling catarrh; and we have all heard of the case lately of the physician and his apprentice boy, who, by leaving his master's house, and lying in the street all night, was cured of a violent catarrh, with which the rest of the family were also affected. This led the Doctor to alter his mode of treating the complaint. Instead of nursing his family, keeping them hot, and sweating them, he exposed them to the air, and the happiest effects followed. I have also put this method into practice, both under my own roof and elsewhere, with similar success.

In speaking or writing on any disease, I would always observe this motto,

"De morbis nil nisi verum."

I am, &c.

*Falmouth, Jan.* 30, 1805. STEPHEN LOWRY, M. D.

